# Enhanced Depth Imaging Optical Coherence Tomography: A New Way Measuring Choroidal Thickness in Pregnant Women

**DOI:** 10.1155/2017/8296574

**Published:** 2017-05-25

**Authors:** Jun Zhang, Huiyun Wang, Qiubo Yu, Qihu Tong, Qinkang Lu

**Affiliations:** ^1^Medical School of Ningbo University, 818 Fenghua Road, Ningbo, Zhejiang, China; ^2^Department of Ophthalmology, Yinzhou Hospital Affiliated to Medical School of Ningbo University, 251 Baizhang East Road, Ningbo, Zhejiang, China; ^3^Department of Obstetrics, Yinzhou Hospital Affiliated to Medical School of Ningbo University, 251 Baizhang East Road, Ningbo, Zhejiang, China

## Abstract

The body changes markedly during pregnancy; each system behaves differently from a nonpregnant state. As the eyes are the only windows to see directly what is going on in the internal environment, more and more researches have been done to explain the association between ocular changes and the physiological and pathological changes during pregnancy. The choroid is one of the critical parts of the eye, providing nutrition. And abnormal choroid may result in ocular dysfunction and visual problems. As the optical coherence tomography develops, a rapid, direct, noninvasive, and nontoxic way is available to obtain the choroid situation of pregnant women, which may explain the mechanism of pregnancy-related eye diseases. This review would summarize relevant original articles published from January 1, 2008 to December 1, 2016 to assess the changes of choroidal thickness (CT) with enhanced depth imaging optical coherence tomography (EDI-OCT) during pregnancy. And the relationship between choroidal thickness changes and pregnancy remains uncertain. To our knowledge, this is the first review of EDI-OCT in assessing the choroidal thickness of the pregnant women.

## 1. Introduction

Pregnancy is a natural state of physiological stress for the body. Each system of the body in a pregnant lady behaves at variation from a nonpregnant state. A complex interplay exists in how the pregnancy affects the eyes and how ocular physiology and pathology may lead to the modification of the management of pregnancy.

The choroid plays a significant part in the physiological function and pathogenesis of eyes: the blood flow per unit weight of choroid is higher than that of any other tissue and choroid acts as the vascular supply for the optic nerve, retinal pigment, outer retina, epithelium, and avascular fovea. So any changes of the choroid may lead to ocular problems.

Traditional ways of measuring choroid have low image resolution and limitation in pregnant women. Although approaches with invasiveness, for example, indocyanine green angiography (ICGA), are still considered as the golden standard to detect lesions in choroid vasculatures of normal eyes as well as diseased eyes, with the development of EDI-OCT, it has been realized to visualize choroid in the body detailedly. And EDI-OCT has been accepted as an essential approach in clinical practice for describing choroidal anatomy three-dimensionally especially in pregnant women.

In the following paragraphs, we will introduce how pregnancy changes ocular systems, methods of measuring, physiology of choroid, and choroidal thickness in gestation.

## 2. How Pregnancy Changes Ocular Systems

**Figure 1:**
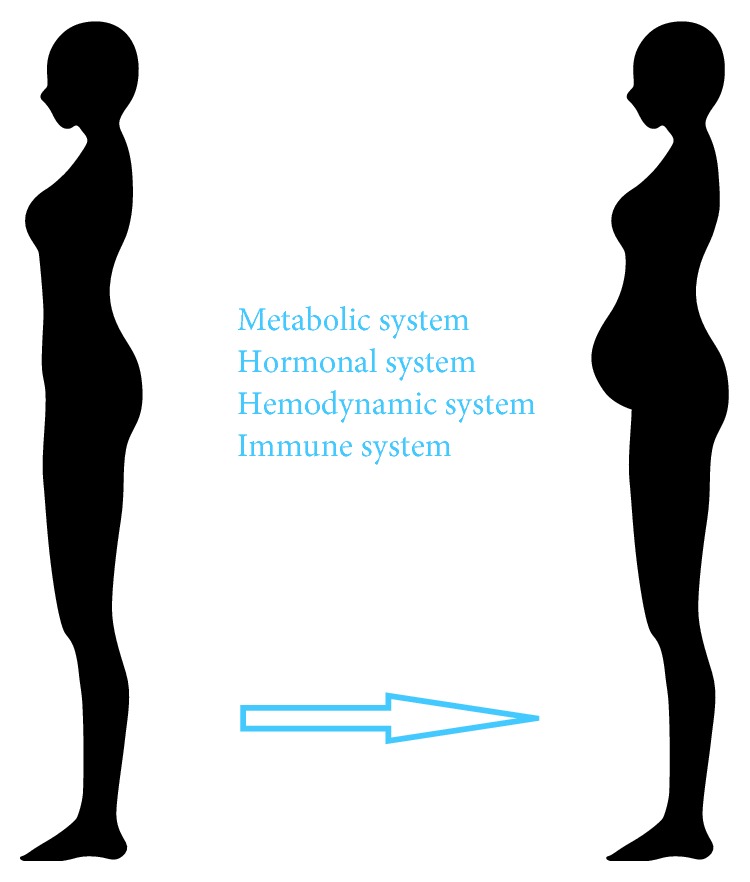
Changes of systems during pregnancy.

**Figure 2:**
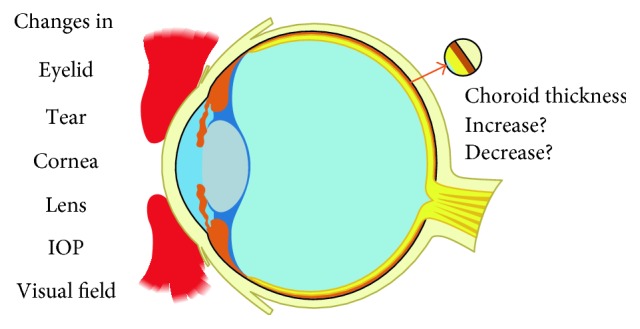
Ocular changes during pregnancy.

Women changes markedly in all systems of the body during normal pregnancy (Figure 1). The body is changing physiologically for the protection of fetus, support for fetal development, and preparation for delivery. The most distinct alterations occur in metabolic, hormonal, and hemodynamic systems. And oestradiol, progesterone, and renin-angiotensin levels increase during pregnancy [1–3]. Oestradiol is known to induce nitric oxide synthases and increase concentrations of nitric oxide, a potent vasodilator, leading to a decreased vascular tone both in the luteal phase of the menstrual cycle and pregnancy [4]. And the change of the renin-angiotensin system leads to increasing blood volume during the first trimester [2, 3] to the last stage of gestation; extracellular body fluid has reached up to 2 L. Systemic vascular resistance also decreases during pregnancy [5, 6]. What is more, the activity of fibrinolysin is decreased and a variety of factors including fibrinogen, plasminogen, factor I, factor V, factor VII, factor IX, and factor X are all increased which leads to a clotting tendency. In the immune system, cellular immunity decreases, but there are no changes in immunoglobins [7]. All of these systems change the organs in the pregnant women and also the eyes (Figure 2). Lots of studies referring to the eyelid, tear, cornea, refraction, lens, intraocular pressure, and visual field have been done previously, except choroid, until recent years. What is more, the association between choroid and other ocular changes during gestation still needs further research. All these changes will be introduced in the following paragraphs, especially choroid.

## 3. Ptosis

It is revealed that ptosis occurs unilaterally in the process of gestation and after normal parturition. It has been accepted that ptosis occurs due to the influences of hormone and body fluid on the levator aponeurosis and could be resolved postpartum [8].

## 4. Tear

Pregnancy affects the tear film physiology resulting in dry eye syndrome [9]. This may attribute to the epidermal growth factor in ductal cells and disruption of lacrimal acinar cells through pregnancy-enhanced immune-reactivity of prolactin and transforming growth factor beta 1 (TGF-*β*1) [9, 10].

## 5. Cornea and Refraction

During pregnancy there may be changes in sensitivity, thickness, or curvature of the cornea. And the decrease of corneal sensitivity becomes more evident at the end of pregnancy [11]. It has been reported that corneal thickness increases during pregnancy in response to corneal oedema [12]. The increase of corneal curvature was also reported by Park et al. [13]. The change of corneal curvature turns to increase significantly during the second and third trimesters and resolve completely after delivery or after stopping of breastfeeding. Changes in corneal thickness may alter the refractive index, which thereby affects refraction. Mehdizadehkashi et al. suggested that the visual acuity alterations start from the first trimester and resume after delivery [14]. A recent research also indicated that pregnancy was inversely associated with myopia development or progression [15]. Many pregnant women develop contact lens intolerance, which may be caused by the increase in corneal curvature or thickness [16]. However, the change may resolve after delivery and pregnant women are advised to wait several weeks postpartum before obtaining a new spectacle prescription.

## 6. Lens

Temporary accommodation loss has been reported with pregnancy and lactation [17]. Consequently, it is recommended to neither prescribe new contact lenses or glasses during the process of gestation and minimally a few months after parturition nor conduct refractive surgeries until the stability of refraction is clear postpartum [18].

## 7. Intraocular Pressure

Intraocular pressure (IOP) decreases as pregnancy advances. Previous studies have shown a statistically significant decrease in IOP during all trimesters of pregnancy compared to nonpregnant women [19]. And the IOP reduction was supposed to be related to various mechanisms: reduced episcleral venous pressure resulted from decreasing total systemic vascular resistance, reduced sclerotic stiffness resulted from elevated tissular resilience, and increased aqueous outflow as well as systemic acidosis in pregnancy [20–22]. And several researches suggest that hypotony causes thickening choroids [23–25], which indicate intraocular pressure may account for the changes of choroidal thickness during gestation.

## 8. Visual Field

Visual field (VF) may change in pregnant women. Akar et al. found that the VF mean threshold sensitivity increased obviously in the third trimester and completely reversed postpartum [26]. The pituitary gland shows physiological enlargement in the process of gestation, which may result in abnormalities, for example, bitemporal concentric visual field defects if the anatomy connection between optic chiasma and pituitary gland is disordered [27].

Choroid also changes during gestation; however, with the special physiology of pregnancy, invasive measuring methods are limited. With technology developing, EDI-OCT provides a new rapid, objective, noninvasive, and nontoxic way to obtain choroid.

## 9. Measuring Methods

Traditionally, indocyanine green angiography, ocular fluorophotometry, ocular angiography, and Doppler ultrasonography were used for assessing ocular findings in gestation [28–33]. Indocyanine green angiography and B-scan ultrasonography are usually performed in the clinical assessment of the choroid, but neither permits accurate cross-sectional imaging. And using indocyanine green or fluorescein is limited during gestation which exhibit teratogenicity as well as safety considerations for feeding with breasts. As all these methods mentioned above have low image resolution and low validity ratio in measurements and differences between observers, a new method which is an objective, reliable, quantitative, and highly sensitive imaging is required for the diagnosis and follow-up of ophthalmic disease in pregnant women.

Optical coherence tomography has provided a rapid, objective, direct, noninvasive, and nontoxic way to obtain high-resolution, real-time images in the retina and retinal pigment epithelium (RPE). And since EDI-OCT was introduced by Spaide et al. in 2008 [34], it has been increasingly used, allowing better understanding of the choroid in health and disease. What is more, a quantitative assessment of overall choroidal anatomy, including volume at the posterior pole and topographic maps of this vascular bed, may do a favor in analyzing choroidal behavior. And four SD-OCT instruments are widely used in measuring the choroidal anatomy: Topcon 3D CT-1000 Mark-II (Topcon Inc., Tokyo, Japan), Optovue RTVue (Optovue Inc., Fremont, CA, USA), Heidelberg Spectralis (Heidelberg Engineering, Germany), and Zeiss Cirrus HD-OCT (Carl Zeiss Meditec Inc., Dublin, CA, USA).

Using EDI-OCT, lots of observations of choroidal thickness in a variety of retina disorders which included polypoid choroid vascular disease, age-depended degeneration of macula, and central serous chorioretinopathy (CSC) have been done. Choroid thickening has been observed in eyes suffering from polypoid choroid vasculopathy (PCV) and CSC while noted in eyes suffering from age-dependent degeneration of macula as well. It was also reported by previous studies that thickened choroid was associated with hyperpermeable choroidal vessels [35, 36]. In 2014, in Turkey, it was used in observing the choroid of pregnant women for the first time [37].

## 10. Physiology of Choroid

The choroid is composed of a vascular network that contributes to ocular nutrition through volume regulation. The blood flowmeter of choroid is higher than that of any other tissue per unit weight [38], and choroid acts as the exclusive vascular supply of retinal pigment epithelium and outer retina, probably part of optical nerve [39] as well as the exclusive source of metabolizing exchanges in the avascular fovea [40]. The choroid also protects the thermal stability of the ocular tissues and removes ocular waste [41].

A structurally and functionally normal choroid is extremely critical to retinal functions. Photoreceptors could dysfunction and even die leading to vision disorder due to abnormality in choroid blood volume or compromise in fluid flow [42]. The structure and thickness of the choroid can be affected by several factors: age, different blood flow, perfusion pressure, ocular pathologies, and systemic diseases [43–47]. In a study of hypertension patients, the author observed that subfoveal choroid was thickened and accumulated subretinal fluid decreased after blood pressure was controlled [48]. The association between systolic pressure and choroid was also varied diurnally in another research [49]. Ocular pathologies, such as choroidal neovascularization (CNV), uveal effusion syndrome (UES), central serous chorioretinopathy (CSC), Vogt-Koyanagi-Harada disease, angioid streaks, and polypoidal choroidal vasculopathy, as well as systemic diseases, including diabetes mellitus, can also affect the choroid [43–47].

It was reported previously that subfoveal choroid was associated with cerebrospinal fluid pressure [50]. Choroidal vascular hyperpermeability [35, 36] and increased serum level [51] are also considered to be associated with choroidal thickening.

In conclusion, the choroid exerts an essential effect on a variable of physiological and pathophysiological conditions and could be affected by diseases that damage vascular system.

## 11. The Choroidal Thickness in Gestation

A lot of transformations could occur in the eyes during gestation such as choroid. To date, 10 studies have been done to discover the association between choroidal thickness and pregnancy [37, 52–60]. And the age of volunteers in all groups was matched. However, the researchers have not come to a conclusion. Characteristics of included studies are shown in Table 1.

## 12. Choroid Thickening

It was proposed that increased CT was related to pregnancy [37, 53, 55–60], and some have suggested that this might relate to the markedly physiology changes in metabolic, hormonal and hemodynamic systems. The choroid performs several important functions such as supplying oxygen and nutrients to the retinal pigment epithelium and the retina up to the inner nuclear layer, temperature regulation, and waste product removal [61]. Thus, a structurally and functionally normal choroidal vasculature is essential for the function of the retina.

In Goktas et al.'s study, 90 healthy pregnant women were included and they were divided into three groups according to their gestational age by the first time: first, second, and third trimesters [57]. By measuring the choroidal thickness at the regions subfoveal, temporal, and nasal to the fovea with EDI-OCT, the author suggested that choroid could be thickened during the second trimester. But with the limitation of pregnancy, ocular blood flow was not measured in this study. The relationship between choroidal thickness and ocular blood flow still needs further research. Kara et al. conducted a study with a larger number of subjects, 100 pregnant women and 100 age-matched nonpregnant women, and significant increase of subfoveal choroidal thickness (SFCT) was also observed [56]. However, the uncertainty between pregnant women and matched nonpregnant women may cause different results. In a research conducted in Portugal [55], using a volumetric analysis described by Chhablani et al. [62], the author evaluated the choroidal structure. And they found that there may be an increase in thickness and volume of maternal choroid in the third trimester of pregnancy. To avoid individual bias, measurements were done during and after pregnancy. And in Turkey, a research suggested that SFCT increased during pregnancy and returned to normal range in three months after delivery [58].

## 13. Uncertain Association

Some studies, however, disagree with the findings above, suggesting that the pregnancy itself does not increase CT [52, 54]. Dadaci et al., for example, found that CT was significantly decreased in healthy pregnant women during the third trimester compared to the first trimester, and the difference between the control group and the pregnant women was not statistically significant(*P* > 0.05) [54]. The inconsistencies were justified by the authors as the small number of the study participants and the limited refraction range. However, the decrease of CT in the third trimester has implications for pregnancy-related ocular diseases, such as CSC, usually occurring at the end of gestation. And the decrease in the choroidal thickness may be explained by the redistribution of blood flow to certain vital organs such as the uterus, kidneys, and skin [7]. And the peak increase of oestrogen and progesterone concentrations and vasoconstriction related to increased adrenoceptor activity can also contribute to the result.

In the recent work of Kim et al., involving 42 eyes from 14 healthy pregnant women, 7 patients with preeclampsia and 14 normal subjects indicated that pregnancy itself did not increase CT [54]. But because of the small number of subjects, more researches of large volunteers are needed.

## 14. Choroid Thickness and Pregnancy-Related Diseases

The correlation between CT evolution and preeclampsia during pregnancy, gestational diabetes mellitus, and central serous chorioretinopathy has not been completely clarified yet.

## 15. Preeclampsia

Preeclampsia occurs in about five percent of puerperae all around the world which results in high mortality rate of puerperae during perinatal stage. And in previous studies, about 25–50% patients with preeclampsia were reported to have visual problems [63]. It has been accepted that a variety of factors cause preeclampsia including oxidative stress injury, heredity, and increasing quantity of autoantibodies type 1 angiotensin II receptor 1 as well as abnormality in the interactions between trophoblasts and decidua which leads to failed invasive activity of trophoplasts into helicine arterioles. Not only that, according to abovementioned contents, multiple organ failure in preeclampsia is mostly caused by hyperpermeable and dysfunctional condition of the endothelium [64]. As a result, cytotoxic and vasogenic oedema cause numerous changes in various organs, such as cerebral, pulmonary, and generalised oedema. Therefore, it is thought that the choroid may have increased interstitial oedema and CT in the same process as other organs.

Some studies have found that the choroid is thinner in preeclampsia pregnant women than in the healthy pregnant women [37, 53, 59], while another found that preeclampsia appears to result in increased CT [54]. Duru et al. and Kim et al. also assessed CT in preeclamptic pregnant women during and after pregnancy [53, 54], and they both arrived at the same conclusion: CT decreased after the parturition of preeclampsia puerperae. In summary, the increase of CT is thought to be caused by increased intracranial pressure in puerperae with preeclampsia or eclampsia and vascular vasospasm, which narrows choroidal vascular structures and contributes to the decrease of CT of the preeclamptic group. What is more, with the limitation of inconvenience and disability during and after delivery, invasive tests such as angiography were not done in these studies, which may discover the blood supply of choroid. And prospective studies with more patients are required.

## 16. Gestational Diabetes Mellitus

Gestational diabetes mellitus (GDM) is a risk factor for the development of type II diabetes and is responsible for both maternal and child morbidity. Professional organizations recommend that diabetes screening for women with GDM should occur around the time of the first postpartum visit [65, 66], and the American Diabetes Association recommends screening at 6–12 weeks after delivery [67]. In a prospective cross-sectional study of GDM conducted by Acmaz et al. [60], choroid, macular, and retinal nerve fiber layer (RNFL) thicknesses were evaluated, and the subjects were divided into 3 groups (group 1—36 pregnant women with GDM; group 2—24 healthy pregnant women; and group 3—38 healthy nonpregnant women). Choroidal thickness turned to be significantly thicker in the healthy pregnant and GDM groups, but no significant difference was observed between the GDM group and the healthy pregnant group. Although it is uncertain whether the change of choroidal thickness is associated with GDM, OCT was suggested for patients with GDM for detection of early retinal changes with GDM.

## 17. Central Serous Chorioretinopathy

It has been reported that CSC may occur in the third trimester of gestation. And studies have been done to find the association between hormonal hypercoagulability, hemodynamic change, pregnancy, and CSC [68, 69]. With the metabolic, hormonal and hemodynamic systems changing during gestation, including increasing level of renin-angiotensin, progesterone, and blood volume which are risk factors reported previously [70], the pregnant women are more likely to have CSC. However, the basis on which CSC develops among gravidae still needs further research. As mentioned above, the change of choroidal thickness and CSC is observed in the nonpregnant women, whether the change of choroid is responsible for CSC in pregnant women still needs further research. Moreover, choroid vascular system could be assessed by enhanced-depth imaging optical coherence tomography without invasiveness or toxicity instead of traditional angiography approach which could be transmitted to fetus through placenta or lactation.

## 18. Conclusion

The study of choroid thickness in pregnant women is a relatively new and attractive field due to recently developed EDI-OCT. Although most of previous studies suggested choroid thickness changes during pregnancy, it is still unrevealed whether the transformations could predict, modulate, cause, or exhibit independency to pregnancy-related ocular diseases, while observations in clinics display inconsistency.

Most observations in clinics support that choroidal thickness increases during gestation, which may account for pregnant-related visual diseases. However, slightly contradictory results, suggesting pregnancy itself may not increase CT. Previous research also found something interesting: mean difference of choroidal thickness was observed between the patients with preeclampsia and pregnant women, and statistical difference existed in different trimesters of gestation.

## 19. Suggestions

In future measurements of choroid thickness of pregnant women, several actions may be taken to reduce bias in many fields, such as diurnal variation, age-related bias, measurement bias, and individual bias.

Several studies have suggested diurnal variation of choroidal thickness [71–74]. And it may be caused by hormone variations based on diel rhythm which could affect systemic blood supply [75]. But in previous studies mentioned above, few measurements were done at the same time of the day. It was reported by Tan et al. in their prospective research that choroid thickness varied significantly in a day [72] and decreased progressively from 9 AM till 5 PM. In Japan, Usui et al. [74] conducted an over 24-hour period study, and it showed similar decreasing trend in choroidal thickness. What is more, increasing vascular supply by overactivated sympathetic system during morning was thought to be embodied in choroidal vascularity which consequently results in thickened choroids [75]. It was suggested by current literature that the time period of measuring choroid thickness was important to both clinical practices and experiments. And in following researches of choroid thickness, measurements should be done at the same time to avoid diurnal variation.

Age acts as one of the most influential elements to choroid thickness. It was reported in previous studies that choriocapillaris diameter, overall luminal area [76, 77], and choroid thickness, as well as vascular density decreased with age. Zengin et al. conducted a cross section which indicated that age exhibited a positive association with SFCT [78]. So, when choroid thickness of pregnant women is evaluated, volunteers may have narrower range in terms of age.

Volume and thickness of choroid were assessed in previous studies. Though measuring the volume of choroid needs more time and cooperation of the patients, it is believed that measurements taken at multiple single points could mislead the global assessment of choroidal anatomy, since the irregularity of the inner chorioscleral border influences the measurement at few sampling points [62, 79]. With technology and software developing, we may evaluate the entire posterior pole instead of few point in our studies.

What is more, researches with long time follow-up of the same pregnant women before, during, and after gestation are suggested, which will avoid individual bias.

Several techniques such as scanning laser ophthalmoscopy (SLO) [80], fundus fluorescein angiography (FFA) [81], laser Doppler flowmetry (LDF) [82–84], laser Doppler velocimetry (LDV) [85], laser speckle flowgraphy (LSFG) [86, 87], pulsatile ocular blood flow (POBF) [88, 89], and color Doppler imaging (CDI) [90, 91] have been used to measure retrobulbar hemodynamics in patients with ocular diseases. CDI is a safe and convenient technique which evaluates erythrocyte velocity in the large ophthalmic vessels, such as ophthalmic artery (OA), central retinal artery (CRA), and nasal and temporal short posterior ciliary arteries (NPCA and TPCA) [92]. And we may apply it to the measurement of the blood flow of choroid of the pregnant women which will assist explaining the relationship between choroidal thickness, ocular vasculature and pregnancy-related eye problems.

Conclusively, owing to the emergence of newest EDI-OCT technologies, choroidal thickness of pregnant women and pregnancy-related ocular diseases have become highly studied clinical entities drawing much attention from researchers. However, the correlation between CT and pregnancy, as well as the effect of choroid in pregnancy-related optical disorders is still unrevealed and future investigations are needed.

## Figures and Tables

**Table 1:** Characteristics of included studies that discovered the association between choroidal thickness and pregnancy.

First author (year)	Location	Groups	Number of patients	Number of eyes	Test time (weeks)	OCT
Sertan Goktas (2014)	Turkey	Pregnant 1	30	30	0–12 w	Heidelberg Engineering
		Pregnant 2	30	30	13–27 w	
		Pregnant 3	30	30	≥28 w	
		Control	30	30	NA	

Mustafa Atas (2014)	Turkey	Pregnant	25	25	≥28 w	Heidelberg Engineering
		Preeclamptic	27	27	NA	
		Control	26	26	≥28 w	

Nihat Sayin (2014)	Turkey	Pregnant	46	46	17–37 w	Cirrus-HD OCT
		Preeclamptic	33	33	16–36 w	
		Control	40	40	NA	

Necip Kara (2014)	Turkey	Pregnant	100	100	15–38	Cirrus-HD OCT
		Control	100	100	NA	

Zeynep Dadaci (2015)	Turkey	Pregnant(S)	27	54	6–8 w	Cirrus-HD OCT
		Pregnant(S)	27	54	32–37 w	
		Control	25	50	NA	

Renata T. Rothwell (2015)	Portugal	Pregnant	12	24	≥28 w	Heidelberg Engineering
		Control	12	24	NA	

Dondu Melek Ulusoy (2015)	Turkey	Pregnant(S)	29	58	≥28 w	Heidelberg Engineering
		Pregnant(S)	29	58	36 w after delivery	
		Control	36	72	NA	

Gokhan Acmaz (2015)	Turkey	Pregnant with GDM	36	NA	≥24 w	Heidelberg Engineering
		Pregnant	24	NA	≥24 w	
		Control	38	NA	NA	

Necati Duru (2016)	Turkey	Pregnant	41	41	≥28 w	Cirrus-HD OCT
		Preeclamptic	32	32	≥28 w	

J. W. Kim (2016)	South Korea	Pregnant	14	14	≥28 w	Heidelberg Engineering
		Preeclamptic	7	7	≥20 w	
		Control	21	21	NA	

NA: not available. Pregnant(S): same pregnant women during and after delivery. Control: matched nonpregnant women.
